# The Nottingham General Hospital

**Published:** 1892-08-13

**Authors:** 


### The Institutional Workshop.

### WITHIN THE HOSPITALS.

### THE NOTTINGHAM GENERAL HOSPITAL.

We have always found heretofore that the further we have travelled south, the worse has been the financial management of corporations and charities. We were under the impression that north of the Thames all financial matters were relatively well and soundly administered. A visit to the Nottingham General Hospital on Monday, a careful inspection of its buildings and arrangements, and an enquiry into its present financial condition, and the action of the Weekly Board, lead us to conclude that we were labouring under a mistake. Evidently finance is not a strong point with the citizens of Nottingham, as represented by the Weekly Board of its General Hospital. A reference to the annual report, and an examination of the abstract of the whole account published on pages 14 and 15, reveal that the annual subscriptions, including arrears for the year ending March 1st, 1892, only amount to £2,111, although the population of Nottingham is upwards of 212,000. Now, when tested by the work done for the hospitals by the citizens of other towns of equal importance in the country, one of two things must be admitted to be true. Either the citizens of Nottingham—a large manufacturing town, containing much machinery, where accidents are frequent, and the need of a well-equipped hospital is urgent—must differ from all their countrymen elsewhere by declining to liberally support the hospital; or the Weekly Board must be held to be blameworthy for not having brought the needs and claims of the General Hospital urgently under the notice of the citizens of Nottingham. Here is an institution, exceptionally well administered, so far as the resident staff are concerned, where the best spirit animates all the workers within the walls, and where the condition and progress of the patients show clearly that they are well cared for, and more than ordinarily comfortable, and yet it fails to obtain from the 212,000 inhabitants of Nottingham even a moderate recognition of the great services it is so efficiently rendering to each and all of the inhabitants. To maintain it adequately and efficiently, with its large out-patient department, and its 145 beds constantly occupied, the moderate sum of £9,000 per annum is required. Towards this, from every source, excluding legacies and investments, the people of Nottingham contributed under £6,400, including receipts from the anniversary and ball. There is, therefore, an annual deficiency of nearly £3,000, and there can be no doubt that a canvass of the city, if aided by the Press, and a representative public meeting, would produce this sum in annual subscriptions in two months' time. At least, from our knowledge of individual citizens and of the action of other provincial towns towards their hospitals, we have every confidence that such a result would follow the adoption of adequate and proper means to awaken the public conscience and to bring the needs of the General Hospital to the notice of the citizens of Nottingham. Is it too much to ask the Mayor to summon a public meeting to lay the facts presented by the annual report before the citizens, and further to express a belief that at least ten Governors of the General Hospital, being the most representative of the citizens, will confer together and take steps to secure the early assembling of a special general board of the Governors under Rule 27, to take into consideration the most urgent question of the present financial condition of one of the oldest and most deservedly popular of our provincial hospitals?

#### Faults of the Weekly Board.

We, of course, base our judgment entirely upon the facts revealed by the published report, and by the present condition and circumstances of the management of this hospital. We have had great experience in the administration of metropolitan and provincial hospitals, and, speaking impartially, and in the best interests of the Nottingham General Hospital, we have with regret to declare that there is abundant evidence of the want of a controlling hand at the Weekly Board, which shall produce a steady, continuous, liberal, fair, wise, and adequate adjustment of the whole machinery of the hospital, whilst care is taken to secure the necessary financial support. We could understand petty and vexatious criticism on the part of inexperienced Governors who might occasionally visit the hospital, and who had no knowledge either of its administration or of hospital management generally. It is, however, quite another matter when members of a Weekly Board continuously act under the mistaken notion that they do not fulfil their duty unless they find fault not only with everything, but with everybody, no matter how good that thing may be, or how zealously and ably that body's duties may be performed. We venture to so speak without any personal knowledge of anybody connected with this hospital. The work of the staff, medical and lay, is exceptionally good. It has to be performed, so far as the older portions of the building are concerned, under conditions which must be most disheartening, and at times most harassing. In these circumstances the Weekly Board, and each individual member of that Board, ought to show not only generous confidence, but warm appreciation of the successful efforts and devoted labours of an able resident staff. We hope that this may be the course of action taken by all the members of the Weekly Board of the Nottingham General Hospital, but we venture to think that the more intelligent of the Governors should take an early opportunity to sharply enquire whether it be so or not. If it be not so, then it is clearly the duty of the Governors to reconstitute the Weekly Board, and to see that the whole administration of the hospital is purified and improved by the election of a Board of Management which shall contain upon it the names of the most representative, able, humane and intelligent of the citizens of Nottingham. Knowledge of all this will not, however, suffice to meet, and to happily terminate the crisis which is impending, we believe, in the affairs of this hospital. The accounts reveal an absence of knowledge and of a sense of proportion to the circumstances of a great institution, as we will now proceed to show a little in detail.

### Defects Revealed in the Accounts.

We have stated already that the income falls short of the expenditure by nearly £3,000 per annum. How is this met? By loans from the bankers and by spending all the legacies received, except, as in the case of Dr. Massey, where the testator requests that they may be invested. Would any man of business in Nottingham who found his income £3,000 less than his expenditure attempt to meet such a deficiency in such an unbusinesslike way? Would he not take care to look his financial position in the face and to evolve some scheme which would increase his income or lessen his expenditure? Let us consider the possibility of reducing the expenditure first. For some reason, which we do not understand, the hospital has no steward and no housekeeper, so that the Matron is at present charged with the duty of looking after the administration of the wards and the nursing of the patients, whilst she has at the same time to manage the commissariat. We have said, as the Weekly Board point out, that the daily average number of in-patients last year was 144½, that is, the greatest number since the hospital was founded 110 years ago. Now, it is perfectly clear that the minimum of inquiry into the systems in force at other similar institutions must prove to any reasonable mind that it is neither fair nor economical to put the burden of the whole of the nursing and of the administration of the wards, in addition to the housekeeping, upon the shoulders of one officer without assistance of any kind. Yet this has been done by the Weekly Board at Nottingham, and we are bound to declare that it reflects the greatest credit upon the officer who has cheerfully and successfully done this work at great personal sacrifice for a number of years, that the average cost of each bed occupied at the present time should be only £56 1s. 9d. This is a very moderate cost, and proves that we were correct in coming to the conclusion that the resident staff are unusually efficient in their various departments. In these circumstances it must be evident that there is no room at the present time for reduction in expenditure at the Nottingham General Hospital, if any proper regard is to be had to the efficiency of its administration. We are therefore thrown back upon the other alternative of an immediate and considerable increase in the income of the institution.

### Birmingham and Nottingham.

Years ago it was ascertained that the charities of Birmingham were maintained by the subscriptions of some 30,000 people, out of a population of 300,000, but this 30,000 contributed at least £1 per head. Nottingham, with its 212 000 inhabitants, contributes less than £6,500, or about sixpence per head, to its hospital. To make up this sixpence, we have had to include every penny which was given through the Hospital Sunday and Hospital Saturday collections. Now, we put it fairly and squarely to every citizen of Nottingham, from the Mayor downwards, and to each of the editors of the Nottingham papers: Is this a state of affairs which is even tolerable when it is bluntly stated in a sentence? We make no doubt that the answer will be an emphatic "No," and that immediate and prompt steps will be taken to bring the facts under the notice of all classes, because a precise knowledge of the facts will make it impossible that the present state of affairs can continue. Unfortunately, the infirmary needs not only £3,000 a year more income, but the time has arrived when the older portion of the buildings should be entirely reconstructed or replaced. This will entail an expenditure of £10,000, and at least another £5,000 is required to provide a new out-patient department, with considerable and suitable accommodation for the nursing staff. The question, therefore, which we put to the citizens of Nottingham is, Will you decline to give £15,000 to make your excellently-administered hospital replete with every modern appliance, and to further contribute in annual subscriptions £3,000 a year more? This represents fifteenpence a head of the population in donations to the Hospital Reconstruction Fund, and an extra contribution to the hospital of threepence per head per annum, making ninepence a head a year all told as a permanent subscription from every citizen. Surely the requirements are moderate enough, and we make no doubt that so wealthy a city will gladly provide the money immediately the facts have been grasped and understood.

### The Present Buildings.

These consist of three main portions—the old general wards, the new wing, and the fever house. Of the old wards little need be said. They were built long ago; they have been adapted as far as possible for hospital purposes; everything has been done by the staff to maintain them in the best possible order, but neither in appearance nor hygienically are they worthy of a great English city. An improvement in appearance can never be secured, because experience shows that it is waste of good money to make the attempt in the case of old adapted buildings used as hospital wards. We were very pleased with the cheerful spirit in which the staff in this part of the hospital do their work, despite the handicap under which they labour, and the fact that the medical and surgical results are so good affords practical evidence of the excellence of the nursing and of the control exercised over the whole establishment by the present resident medical staff and the Matron. The new wing presented a striking contrast to the old and central building. It contains two really excellent wards, the condition of which left literally nothing to be desired. Everything was in apple-pie order; the furniture, lavatories, and baths offer little to criticise, whilst the neatness and order which an examination of the cupboards revealed was most encouraging and satisfactory. We would venture to suggest that the Mayor and other representative citizens, and also the editors of the local papers, should personally visit the hospital, and go first of all into the so-called new wing—built twelve years ago—and then into the old and central portion, because such a step would afford them an object-lesson, which must result, we are confident, in the speedy reconstruction of those portions of the hospital buildings which urgently call for entire renovation. Like the new wing, the fever house was excellently kept, and we could not help feeling that if it was our fate to be striken down by fever, we could not wish to find ourselves in a more comfortable place than this branch of the Nottingham General Hospital undoubtedly is. Only a few of the beds were occupied at the time of our visit, but every bed in every ward was ready for occupation, and the thorough order throughout the fever house was another proof of the excellence of the internal management which prevails at Nottingham to-day. We purposely avoid saying anything about the out-patient department, as the Committee in their report admit "the necessity of improving the accommodation, which at present causes discomfort both to the out-patients and the medical gentlemen who attend them."

Minor Suggestions.

Exactly opposite the General Hospital stands the Free Hospital for Sick Children. In one division of the old building we were surprised to find that many of the beds were occupied by children, the majority of whom were under five years of age. In one bed there were two babies, and altogether in this division we counted five very young children. There were also small children in other divisions. Now, how is it that these young children are admitted to the adult wards of the General Hospital, and that the beds contain so large a proportion of children, seeing that there is a children's hospital across the way, which ought surely to contain sufficient accommodation to admit all these cases? Be this as it may, however, it is quite certain that the Weekly Board and medical staff would be well advised, should they find it necessary or desirable to continue the practice of admitting children, and especially young children, that one of the larger wards should be set apart exclusively for their accommodation. It is bad enough to mix children with adult patients in a large general ward, but it is worse to have a number of small wards on the same floor, and to scatter the children through them all, seeing that it is impossible for the Sister, with the assistance of one charge nurse and one probationer only, to readily attend to their requirements. We would further call the attention of the Committee to the condition of the gas fittings. This is a small matter, but it requires attention. The globes in the ventilated gasaliers have been apparently broken, and not replaced. Now, if it was worth while to incur the expense of putting up the patent gasaliers to be found in the Nottingham General Hospital, it is certainly worth while that they should be provided with the necessary globes to enable them to do their work efficiently, and so to help to purify the ward air. It is further desirable in all hospitals that provision should be made for the adequate exercise and recreation of the nursing staff. At the present time the nurses have no opportunity of getting exercise by playing lawn tennis, although there is an excellent lawn in the garden, which might be given up to them during certain hours each day, or on certain days in each week. We ascertained that this would not interfere with the use of the lawn tennis court by the resident medical staff, and we shall hope to hear that arrangements have been made to utilise the ground for the benefit of all the staff without a moment's delay. We would further draw attention to the fact that there is not at the present time a library for the use of the nurses, and we would suggest that the ladies of Nottingham might usefully take this matter in hand, and see that a good supply of books is forthcoming for the nurses' use. We hope that our remarks will be taken in good part. We assure all concerned that we shall be very happy to co-operate to bring about the necessary alterations, both in the building and system of administration, and that any information or help which we can render to the Governors or the Weekly Board, or the citizens of Nottingham, in order to make the General Hospital replete with every modern appliance, and in every sense worthy of the town in which it is situated, will be gladly, nay thankfully, rendered.

